# School Performance of Preterm-Born Children After Intraventricular Hemorrhage

**DOI:** 10.1001/jamanetworkopen.2025.47584

**Published:** 2025-12-11

**Authors:** Philippa Rees, Mithilesh Dronavalli, Ben Carter, Michelle Dickson, Charles Green, Kate Lawler, Evelyn Lee, Hannah Uebel, Chris Gale, Ju Lee Oei

**Affiliations:** 1 Population Policy and Practice, University College London Institute of Child Health, London, United Kingdom; 2 Translational Health Research Institute, Western Sydney University, Penrith, New South Wales, Australia; 3 Department of Biostatistics and Health Informatics, Institute of Psychiatry, Psychology and Neuroscience, King’s College London, London, United Kingdom; 4 The Poche Centre for Indigenous Health, Faculty of Medicine and Health, The University of Sydney, Camperdown, New South Wales, Australia; 5 Alpha Maxx Healthcare, Memphis, Tennessee; 6 Discipline of Paediatrics and Child Health, School of Clinical Medicine, University of New South Wales, Kensington, New South Wales, Australia; 7 Centre for Social Research in Health, University of New South Wales, Kensington, New South Wales, Australia; 8 Centre for Economic Impacts of Genomic Medicine, Macquarie University, North Ryde, New South Wales, Australia; 9 Neonatal Medicine, School of Public Health, Imperial College London, London, United Kingdom; 10 Centre for Paediatrics and Child Health, Imperial College, London, London, United Kingdom; 11 Department of Newborn Care, Royal Hospital for Women, Randwick, New South Wales, Australia

## Abstract

**Importance:**

Intraventricular hemorrhage (IVH) is a significant complication of preterm birth, affecting approximately 20% of preterm infants. Despite its prevalence, the effect of IVH beyond the impact of prematurity alone has been scarcely studied beyond early childhood, posing an important knowledge gap.

**Objective:**

To investigate the association of IVH with national school performance throughout childhood to adolescence.

**Design, Setting, and Participants:**

This population-based cohort study included all very preterm infants (<32 weeks’ gestation) and full-term infants (≥37 weeks’ gestation) born in New South Wales, Australia, between January 1, 2007, and December 31, 2013. Cohorts were very preterm children with low-grade (grades 1-2) or high-grade (grades 3-4) IVH, very preterm controls without IVH, and full-term controls. Analyses were conducted from January 30 to September 18, 2024.

**Exposure:**

IVH grade 1 to 4.

**Main Outcomes and Measures:**

The primary outcome was overall performance on standardized national school assessments at age 8 to 9, 10 to 11, and 12 to 13 years, including adjusted mean differences (AMDs) in *z* scores between children with IVH and very preterm controls. Secondary outcomes were domain-specific performance in reading, writing, spelling, grammar, and numeracy and whether children met the national minimum standards overall and for each domain. Academic trajectories were also compared by group.

**Results:**

This study included 408 189 children: 557 with low-grade IVH, 85 with high-grade IVH, 2557 very preterm controls without IVH, and 404 990 full-term controls. Children with low-grade IVH performed similarly to preterm controls at age 8 to 9 years (AMD in overall academic *z* score, −0.06; 95% CI, −0.14 to 0.03), 10 to 11 years (AMD, −0.09; 95% CI, −0.21 to 0.03), and 12 to 13 years (AMD, −0.04; 95% CI, −0.24 to 0.16). Children with grade 2 IVH (n = 145), however, performed significantly worse than very preterm controls at age 8 to 9 years (AMD, −0.20; 95% CI, −0.36 to −0.04). Children with high-grade IVH performed significantly worse than very preterm controls at age 8 to 9 years (AMD, −0.50; 95% CI, −0.71 to −0.30), 10 to 11 years (AMD, −0.59; 95% CI, −0.85 to −0.34), and 12 to 13 years (AMD, −0.61; 95% CI, −1.05 to −0.17). Numeracy was a consistently weak domain for children with high-grade IVH throughout school age (eg, at age 8 to 9 years, AMD in the numeracy *z* score compared with very preterm controls was −0.49 [95% CI, −0.70 to −0.28]). Differences in academic trajectories between groups remained fixed with increasing age; however, all groups showed improvement over time (eg, adjusted β for very preterm children, 32.3 [95% CI, 31.2-33.5]; children with low-grade IVH, 31.5 [95% CI, 29.0-34.0]; high-grade IVH, 30.2 [95% CI, 24.1-36.4]).

**Conclusions and Relevance:**

In this cohort study, the association of low-grade IVH with worse school performance appeared limited to children with grade 2 IVH. Children with high-grade IVH consistently showed poorer academic performance into adolescence than their peers born very preterm without IVH. Nevertheless, very preterm children, regardless of IVH grade, demonstrated academic progress over time, underscoring the need for ongoing educational support to help them to realize their full potential.

## Key Points

**Question:**

What is the association between intraventricular hemorrhage (IVH) in very preterm infants (<32 weeks’ gestation) and school performance?

**Findings:**

In this population-based cohort study of 408 189 children in Australia, very preterm children with grade 1 to 2 IVH performed similarly to those without IVH at school age, although grade 2 IVH was associated with poorer academic outcomes. Survivors with grade 3 to 4 IVH consistently achieved lower scores, particularly in numeracy, but most still met the minimum standard for national assessments.

**Meaning:**

The findings suggest very preterm infants with high-grade, low-grade, and no IVH have parallel trajectories of academic improvement with age, highlighting the importance of ongoing academic support.

## Teaser

This cohort study investigates whether intraventricular hemorrhage in children born at less than 32 weeks’ gestation is associated with school performance at age 8 to 13 years.

## Introduction

Preterm birth presents a significant challenge worldwide, affecting approximately 10% of infants and leading to serious neurologic complications.^1,2,3,4^ Despite advances in neonatal care, these complications continue to pose a public health challenge.^1,5,6^ The Global Burden of Disease Study highlighted a 29.2% increase in years lived with disability due to neurologic issues from prematurity between 1990 and 2021, underscoring the growing burden of these conditions.^7^

Despite a widespread focus on neuroprotection in the preterm population, the incidence of intraventricular hemorrhage (IVH) remains stable at approximately 20%.^1,5,6,8,9,10^ This is problematic given the immaturity of the preterm brain and its vulnerability to long-term neurodevelopmental sequelae.^6^ Historically, low-grade IVH (grade 1-2) has been thought to have minimal impact on neurodevelopment; however, recent evidence indicates a significant association with moderate-severe neurodevelopmental impairment (NDI) (adjusted odds ratio [AOR], 1.35; 95% CI, 1.05-1.75).^6,11^ This was reinforced by a recent population study reporting a reduction in survival without severe NDI following low-grade IVH, primarily driven by grade 2 IVH.^9^ While low-grade IVH has been associated with cerebral palsy and lower IQ scores in school-aged children, these findings originated from nonrepresentative historical cohorts.^12,13,14,15,16,17^ Similarly, though high-grade IVH (grade 3-4) is associated with severe NDIs across cognitive, motor, communication, visual, and hearing domains at 2 years of age, contemporary data on school-age outcomes are limited.^11,16,18,19,20,21^

Consequently, a significant research gap exists regarding the functional outcomes at school age of very preterm children (born at <32 weeks’ gestation) with either low- or high-grade IVH. School performance is a key metric of childhood functional ability across several developmental domains, and it predicts future adult success.^22^ Additionally, the standardized and longitudinal nature of school-performance data provides useful measures of children’s trajectories individually and compared with their peers. Therefore, this population-based study focused on exploring the academic performance and trajectories of very preterm children with differing grades of IVH compared with their very preterm peers without IVH and term-born (≥37 weeks’ gestation) peers to investigate the association of IVH, both high-grade and low-grade, with national school performance throughout childhood. This is important for differentiating the effects of IVH from the influence of prematurity on children’s outcomes.

## Methods

### Study Design

This retrospective cohort study involved all very preterm infants born in New South Wales, Australia, and admitted to a neonatal unit between January 1, 2007, and December 31, 2013. The Centre for Health Record Linkage used probabilistic matching of personal identifiers—including name, date of birth, address, and hospital record number—to link infants across datasets and assign them a unique identifier. No identifiable information was accessible to the researchers. The New South Wales Population and Health Services and Australian Capital Territory Department of Health research ethics committees, the Aboriginal Health and Medical Research Council, and the Australian education sectors granted ethical approval and waived informed consent because data were anonymized. This study followed the Strengthening the Reporting of Observational Studies in Epidemiology (STROBE) reporting guideline for cohort studies.

The preterm cohorts for this study were established within the Neonatal Intensive Care Units (NICU) Data Collection dataset and linked to several additional Australian datasets to obtain covariate and outcome data: the Perinatal Data Collection (PDC), Admitted Patient Data Collection (APDC), Australian Bureau of Statistics Causes of Death, and National Assessment Program—Literacy and Numeracy (NAPLAN) databases (eTable 1 in Supplement 1). A cohort of full-term controls (born at ≥37 weeks of gestation) was also derived from the New South Wales PDC and APDC datasets.

### Participants

Any infant born between January 1, 2007, and December 1, 2013, in New South Wales at less than 32 weeks’ gestation (when the risk of IVH is greatest) was included. Infants with grade 3 or 4 IVH were classified as having high-grade IVH and those with grade 1 or 2 as having low-grade IVH. Preterm infants born at less than 32 weeks of gestation during the same period but without IVH were included as very preterm controls. IVH was diagnosed on standardized cranial ultrasonography assessments, which are routinely conducted by neonatologists, radiologists, or sonographers for preterm infants in the first week of life.^8^ The highest recorded grade of IVH as per the Papile classification was used.^23^ Full-term infants born at 37 or more weeks of gestation during the same period and without NICU admission formed the full-term control group for mean NAPLAN score and trajectory analyses.

### Covariates

Key covariates—sex, gestational age, birth weight *z* score, multiples birth, receipt of antenatal magnesium sulfate, and receipt of antenatal steroids—were extracted from NICU records. Additional variables—Indigenous status, socioeconomic decile, Accessibility/Remoteness Indicator of Australia (ARIA) score, and maternal smoking status—were derived from PDC or APDC records. Parental educational level was available in the NAPLAN dataset. Covariates for adjustment were specified a priori in a directed acyclic graph (eFigure 1 in Supplement 1).

### Outcome Measures

The primary outcome was total school performance *z* score across all 5 NAPLAN domains at age 8 to 9 years (school grade 3), 10 to 11 years (school grade 5), and 12 to 13 years (school grade 7). Secondary outcomes at the same ages and grades were (1) NAPLAN reading, writing, spelling, grammar, and numeracy *z* scores; (2) the proportion of children meeting national minimum standards (NMS) overall and by NAPLAN domain; and (3) trajectories of school performance overall and by NAPLAN domain.

### Statistical Analysis

A complete case analysis was conducted, including all surviving participants with a NAPLAN record. The characteristics of very preterm infants with and without IVH were described and compared using χ^2^ tests for categorical variables, *t* tests for normally distributed continuous variables, and Mann-Whitney *U* tests for nonnormally distributed continuous variables. Total mean scores across the 5 NAPLAN domains were calculated at age 8 to 9 years (school grade 3), 10 to 11 years (school grade 5), and 12 to 13 years (school grade 7). These scores were standardized within the very preterm population at each grade and across test years to create total NAPLAN *z* scores and *z* scores for each of the 5 NAPLAN domains assessed. Children without an attainment score, who were designated as not meeting the NMS, were allocated the lowest score for their test year as per a previous study.^24^ This approach was used to account for children with disabilities who were exempt from testing and therefore had missing attainment scores that were not missing at random. These children were classed as below the NMS by default. Treating their missing outcomes in this way ensured that children with special needs or testing exemptions were included.

Linear regression was used to determine the crude mean differences (MDs) and adjusted MDs (AMDs) in *z* scores between children with high-grade IVH, children with low-grade IVH, and very preterm controls. Logistic regression was used to estimate the crude ORs and AORs for meeting the NMS overall as well as for each of the 5 specific NAPLAN domains for children with high-grade IVH, children with low-grade IVH, and very preterm controls. The 95% CIs were calculated for all point estimates.

The mean total and domain-specific NAPLAN scores for children with high-grade IVH, children with low-grade IVH, very preterm controls, and full-term controls were analyzed at ages 8 to 9 years, 10 to 11 years, and 12 to 13 years. Longitudinal trajectories in mean academic scores over time for these 4 groups were analyzed using hierarchical growth curve modeling, which uses all available data points from each participant. This approach enabled the estimation of the intercept, indicating initial academic performance at 8 to 9 years, and the slope (β), indicating the rate of change in scores from ages 8 to 13 years (grades 3 to 7). By including a group × time interaction term, the model captured the varying academic trajectories (slopes) based on IVH severity while controlling for specified confounders. *P* < .05 was used to determine significance. Analyses were performed from January 30 to September 18, 2024, using Stata, version 18 (StataCorp LLC). Outcomes were compared by individual grade of IVH in subgroup analyses where possible.

## Results

Between 2007 and 2013, 5481 very preterm and 622 351 full-term neonates were born in New South Wales (eFigure 2 in Supplement 1). The cohort of 408 189 surviving children with a NAPLAN record (2016-2019 and 2021) included 85 children with high-grade IVH, 557 with low-grade IVH, 2557 very preterm controls, and 404 990 full-term controls.

There were differences between study groups. The mean (SD) gestational age was 28 (2.2) weeks for children with low-grade IVH, 27 (2.1) weeks for children with high-grade IVH, 29 (1.9) weeks for very preterm controls, and 39.3 (1.2) weeks for term-born controls (Table 1). Males were more commonly represented in the groups with low-grade IVH (females, 244 [43.8%]; males, 313 [56.2%]) and high-grade IVH (females, 29 [34.1%]; males, 56 [65.9%]) than in the very preterm control group (females, 1241 [48.5%]; males, 1316 [51.5%]) and full-term control group (females, 200 182 [49.4%]; males, 204 721 [50.5%]). Those with IVH were less likely to have received antenatal steroids or antenatal magnesium sulfate. Most very preterm survivors born from 2007 to 2010 had a NAPLAN record (2504 of 2881 [86.9%]), with no significant differences between IVH cases and very preterm controls. The numbers with a NAPLAN record were lower in later years due to the COVID-19 pandemic resulting in the cancellation of the NAPLAN assessments in 2020 (eFigure 2 in Supplement 1).

**Table 1.** Characteristics of the Included Population

Characteristic	Children^a^		
	Very preterm controls (n = 2557)	Low-grade IVH (n = 557)	High-grade IVH (n = 85)
Gestational age, mean (SD), wk	29 (1.9)	28 (2.2)	27 (2.1)
Birth weight *z* score, mean (SD)	0.0 (1.0)	0.2 (1.0)	0.2 (0.8)
Sex			
Female	1241 (48.5)	244 (43.8)	29 (34.1)
Male	1316 (51.5)	313 (56.2)	56 (65.9)
Multiples birth	758 (22.9)	129 (23.2)	18 (21.2)
Receipt of antenatal magnesium sulfate	1602 (62.7)	324 (58.2)	46 (54.1)
Receipt of antenatal steroids	2337 (91.4)	489 (87.8)	71 (83.5)
SES decile, mean (SD)	5.1 (2.7)	5.0 (2.7)	4.8 (2.8)
Indigenous status	285 (11.3)	62 (11.2)	11 (13.1)
ARIA score, mean (SD)^b^	10.3 (0.6)	10.3 (0.6)	10.3 (0.5)
Maternal smoking	540 (21.3)	122 (21.9)	16 (19.1)
Maternal age, mean (SD), y	30.5 (6.2)	29.3 (6.3)	29.4 (6.3)
Parent completed high school or higher	1943 (81.6)	420 (79.9)	66 (82.5)
Missing attainment score and not meeting NMS at school grade 3	154 (6.0)	51 (9.2)	18 (21.2)

Abbreviations: ARIA, Accessibility/Remoteness Index of Australia; IVH, intraventricular hemorrhage; NMS, national minimum standards; SES, socioeconomic status.

^a^ Data are presented as number (percentage) of children unless otherwise indicated. Very preterm indicates gestational age less than 32 weeks.

^b^ Score range of 0 to 15, with higher scores indicating increasing remoteness.

Children with high-grade IVH were more likely to have missing attainment scores and therefore to be classified as not meeting the NMS (18 [21.2%]) compared with those with low-grade IVH (51 [9.2%]) and very preterm controls (154 [6.0%]) (Table 1). The impact of imputing missing attainment scores for these children is shown in eTable 2 in Supplement 1.

### Low-Grade IVH

Children born very preterm with low-grade IVH had consistently numerically lower total NAPLAN *z* scores (AMD, −0.06; 95% CI, −0.14 to 0.03) and domain-specific *z* scores compared with very preterm controls at age 8 to 9 years, although differences were not clinically or statistically significant and did not reach statistical significance after adjusting for covariates (Table 2). The overall rate of meeting the NMS at age 8 to 9 years was similar for those with low-grade IVH (386 of 552 with NAPLAN data [69.9%]) and very preterm controls (1914 of 2525 with NAPLAN data [75.8%]) (AOR, 0.85; 95% CI, 0.68-1.08) (Table 3). However, after disaggregating by IVH grade, children with grade 2 IVH had significantly lower total *z* scores at age 8 to 9 years compared with very preterm controls (AMD, −0.20; 95% CI, −0.36 to −0.04), while no difference was found for children with grade 1 IVH compared with very preterm controls (AMD, −0.01; 95% CI, −0.11 to 0.08) (eTable 9 in Supplement 1).

**Table 2.** Linear Regression Results for School Performance of Children With High- and Low-Grade IVH Compared With Very Preterm Controls at Age 8 to 9 Years in School Grade 3^a^

NAPLAN domain	Low-grade IVH		High-grade IVH	
	MD (95% CI)	AMD (95% CI)^b^	MD (95% CI)	AMD (95% CI)^b^
Overall	−0.13 (−0.22 to −0.04)	−0.06 (−0.14 to 0.03)	−0.56 (−0.77 to −0.35)	−0.50 (−0.71 to −0.30)
Reading	−0.11 (−0.2 to −0.02)	−0.05 (−0.14 to 0.04)	−0.50 (−0.71 to −0.29)	−0.47 (−0.68 to −0.26)
Writing	−0.10 (−0.19 to −0.01)	−0.03 (−0.12 to 0.06)	−0.52 (−0.73 to −0.31)	−0.43 (−0.64 to −0.22)
Spelling	−0.10 (−0.19 to −0.01)	−0.03 (−0.13 to 0.06)	−0.44 (−0.65 to −0.23)	−0.42 (−0.64 to −0.21)
Grammar	−0.14 (−0.23 to −0.05)	−0.08 (−0.17 to 0.01)	−0.53 (−0.74 to −0.32)	−0.47 (−0.68 to −0.25)
Numeracy	−0.14 (−0.23 to −0.05)	−0.06 (−0.15 to 0.03)	−0.54 (−0.75 to −0.33)	−0.49 (−0.70 to −0.28)

Abbreviations: AMD, adjusted mean difference; IVH, intraventricular hemorrhage; MD, mean difference; NAPLAN, National Assessment Program—Literacy and Numeracy.

^a^ Five infants with low-grade IVH and 32 very preterm controls did not have NAPLAN results at age 8 to 9 years (school grade 3) but did have school grade 5 or 7 results.

^b^ Adjusted for gestational age, birth weight *z* score, sex, receipt of antenatal magnesium sulfate, receipt of antenatal steroids, multiples birth, Indigenous status, Accessibility/Remoteness Index of Australia score (rurality), socioeconomic status, maternal age, maternal smoking in pregnancy, parental educational level, test year, and age at test.

**Table 3.** Logistic Regression Results for School Performance Above the National Minimum Standard Among Children With High- and Low-Grade IVH Compared With Very Preterm Controls at Age 8 to 9 Years in School Grade 3^a^

NAPLAN domain	Children, No. (%)			Low-grade IVH		High-grade IVH	
	Very preterm controls (n = 2525)^b^	Low-grade IVH (n = 552)	High-grade IVH (n = 85)	OR (95% CI)	AOR (95% CI)^c^	OR (95% CI)	AOR (95% CI)^c^
Overall	1914 (75.8)	386 (69.9)	49 (57.7)	0.74 (0.61 to 0.91)	0.85 (0.68 to 1.08)	0.43 (0.28 to 0.67)	0.47 (0.29 to 0.78)
Reading	2200 (87.1)	460 (83.3)	61 (71.8)	0.74 (0.57 to 0.95)	0.80 (0.60 to 1.06)	0.38 (0.23 to 0.61)	0.38 (0.22 to 0.64)
Writing	2221 (88.0)	468 (84.8)	58 (68.2)	0.76 (0.60 to 0.99)	0.83 (0.62 to 1.11)	0.29 (0.18 to 0.47)	0.30 (0.18 to 0.51)
Spelling	2160 (85.5)	449 (81.3)	56 (65.9)	0.74 (0.58 to 0.94)	0.84 (0.64 to 1.10)	0.33 (0.21 to 0.52)	0.33 (0.20 to 0.55)
Grammar	2171 (86.0)	455 (82.4)	59 (69.4)	0.76 (0.6 to 0.98)	0.86 (0.65 to 1.13)	0.37 (0.23 to 0.59)	0.40 (0.23 to 0.68)
Numeracy	2166 (85.8)	444 (80.4)	57 (68.1)	0.68 (0.54 to 0.86)	0.80 (0.61 to 1.04)	0.34 (0.21 to 0.54)	0.37 (0.22 to 0.62)

Abbreviations: AOR, adjusted odds ratio; IVH, intraventricular hemorrhage; NAPLAN, National Assessment Program—Literacy and Numeracy; OR, odds ratio.

^a^ Five infants with low-grade IVH and 32 very preterm controls did not have NAPLAN results at age 8 to 9 years (school grade 3) but did have school grade 5 or 7 results.

^b^ Very preterm indicates gestational age less than 32 weeks.

^c^ Adjusted for gestational age, birth weight *z* score, sex, receipt of antenatal magnesium sulfate, receipt of antenatal steroids, multiples birth, Indigenous status, Accessibility/Remoteness Index of Australia score (rurality), socioeconomic status, maternal age, maternal smoking in pregnancy, parental educational level, test year, and age at test.

Very preterm children with low-grade IVH who had NAPLAN scores had lower total NMS attainment scores (MD, −0.17; 95% CI, −0.29 to −0.05) and domain-specific NAPLAN scores compared with very preterm controls at age 10 to 11 years, but again, these were not significantly different from scores of very preterm controls after adjusting for confounders (AMD, −0.09; 95% CI, −0.21 to 0.03). Rates of meeting the NMS at age 10 to 11 years were similar for those with low-grade IVH (202 of 297 [68.0%]) and very preterm controls (1123 of 1538 [73.0%]) (AOR, 0.89; 95% CI, 0.65-1.21), although those with low-grade IVH were less likely than the very preterm controls to meet the NMS for the reading (AOR, 0.68; 95% CI, 0.47-0.98) and grammar (AOR, 0.71; 95% CI, 0.50-1.00) assessments (eTable 10 in Supplement 1). No statistically significant differences at age 12 to 13 years (school grade 7) were detected between those with low-grade IVH and very preterm controls in total scores (AMD, −0.04; 95% CI, −0.24 to 0.16) (Table 4) or rates of meeting the NMS.

**Table 4.** Linear Regression of School Performance at 10 to 11 Years and 12 to 13 Years Among Children With High- and Low-Grade IVH Compared With Very Preterm Controls^a^

NAPLAN domain	Low-grade IVH		High-grade IVH	
	MD (95% CI)	AMD (95% CI)^b^	MD (95% CI)	AMD (95% CI)^b^
**10-11 y (school grade 5)**				
Total	−0.17 (−0.29 to −0.05)	−0.09 (−0.21 to 0.03)	−0.62 (−0.88 to −0.35)	−0.59 (−0.85 to −0.34)
Reading	−0.16 (−0.28 to −0.03)	−0.10 (−0.21 to 0.02)	−0.67 (−0.93 to −0.40)	−0.65 (−0.91 to −0.40)
Writing	−0.14 (−0.26 to −0.02)	−0.07 (−0.18 to 0.05)	−0.53 (−0.80 to −0.27)	−0.50 (−0.75 to −0.25)
Spelling	−0.16 (−0.28 to −0.04)	−0.09 (−0.21 to 0.03)	−0.53 (−0.79 to −0.26)	−0.52 (−0.78 to −0.26)
Grammar	−0.17 (−0.30 to −0.05)	−0.10 (−0.22 to 0.02)	−0.50 (−0.76 to −0.23)	−0.45 (−0.71 to −0.19)
Numeracy	−0.14 (−0.26 to −0.01)	−0.05 (−0.17 to 0.07)	−0.55 (−0.81 to −0.29)	−0.56 (−0.82 to −0.30)
**12-13 y (school grade 7)**				
Total	−0.20 (−0.40 to 0.01)	−0.04 (−0.24 to 0.16)	−0.61 (−1.04 to −0.18)	−0.61 (−1.05 to −0.17)
Reading	−0.18 (−0.39 to 0.02)	−0.04 (−0.24 to 0.17)	−0.48 (−0.92 to −0.05)	−0.43 (−0.89 to 0.03)
Writing	−0.12 (−0.32 to 0.09)	0.02 (−0.19 to 0.22)	−0.54 (−0.98 to −0.11)	−0.49 (−0.95 to −0.03)
Spelling	−0.19 (−0.40 to 0.01)	−0.06 (−0.26 to 0.13)	−0.58 (−1.01 to −0.15)	−0.66 (−1.09 to −0.23)
Grammar	−0.21 (−0.42 to −0.01)	−0.09 (−0.29 to 0.11)	−0.56 (−0.99 to −0.13)	−0.57 (−1.02 to −0.13)
Numeracy	−0.18 (−0.38 to 0.03)	−0.00 (−0.21 to 0.20)	−0.59 (−1.04 to −0.15)	−0.65 (−1.12 to −0.19)

Abbreviations: AMD, adjusted mean difference; IVH, intraventricular hemorrhage; MD, mean difference; NAPLAN, National Assessment Program—Literacy and Numeracy.

^a^ Very preterm indicates gestational age less than 32 weeks.

^b^ Adjusted for gestation, birth weight *z* score, sex, receipt of antenatal magnesium sulfate, receipt of antenatal steroids, multiples birth, Indigenous status, Accessibility/Remoteness Index of Australia score (rurality), socioeconomic status, maternal age, maternal smoking in pregnancy, parental educational level, test year, and age at test.

### High-Grade IVH

Very preterm children with high-grade IVH had consistently lower total and domain-specific academic scores compared with their very preterm peers without IVH at age 8 to 9 years (Table 2). This difference remained clinically and statistically significant after adjustment (eg, overall NAPLAN AMD, −0.50; 95% CI, −0.71 to −0.30). Children with high-grade IVH performed particularly poorly in numeracy compared with very preterm controls (AMD, −0.49; 95% CI, −0.70 to −0.28). Children with high-grade IVH were also less likely to meet the NMS at age 8 to 9 years compared with preterm controls (AOR, 0.47; 95% CI, 0.29-0.78); however, most of the children with high-grade IVH (49 [57.7%]) did meet the NMS.

These differences persisted at age 10 to 11 years, where children with high-grade IVH continued to have significantly lower total attainment scores (AMD, −0.59; 95% CI, −0.85 to −0.34) and domain-specific academic scores compared with very preterm controls. This was most marked in reading (AMD, −0.65; 95% CI, −0.91 to −0.40) and numeracy (AMD, −0.56; 95% CI, −0.82 to −0.30). The significant difference in rates of meeting the NMS for the total NAPLAN assessment (AOR, 0.32; 95% CI, 0.17-0.60) and domain-specific assessments also persisted, with only half of those with high-grade IVH (27 of 54 [50.0%]) meeting the NMS for the total NAPLAN at age 10 to 11 years.

At age 12 to 13 years, these differences remained. Children with high-grade IVH at this age performed significantly worse than very preterm controls in total attainment scores (AMD, −0.61; 95% CI, −1.05 to −0.17) and domain-specific scores, especially numeracy (AMD, −0.65; 95% CI, −1.12 to −0.19) and spelling (AMD, −0.66; 95% CI, −1.09 to −0.23). Significant differences in rates of meeting the NMS overall (AOR, 0.16; 95% CI, 0.05-0.49) and domain-specific NMS also persisted; for example, most children with high-grade IVH did not meet the NMS for reading at age 12 to 13 years (14 of 21 [66.7%]).

### Trajectories

A consistent stepwise reduction in mean overall attainment scores was observed across groups, with full-term controls exhibiting the highest scores, followed by very preterm controls, children with low-grade IVH, and children with high-grade IVH (Figure and eTable 3 in Supplement 1). Similar trajectories were observed in the mean scores for reading, writing, spelling, grammar, and numeracy for each group at each school grade (Figure and eTables 4-8 in Supplement 1).

**Figure.**
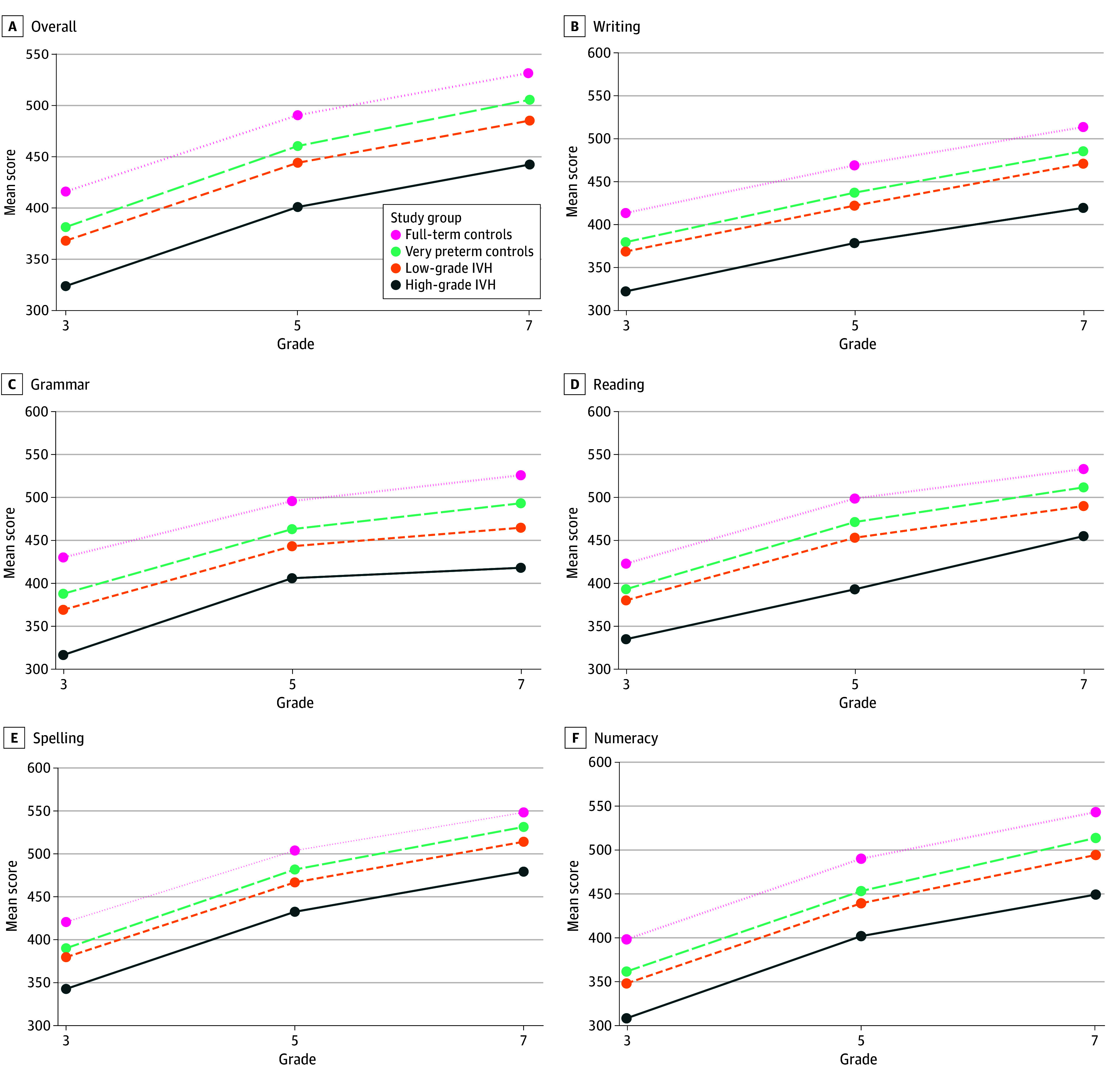
Mean National Assessment Program—Literacy and Numeracy Score Trajectories of Children Born From 2007 to 2013 Very preterm indicates gestational age of less than 32 weeks; full-term, gestational age of 37 weeks or greater.

Differences in school performance scores remained consistent and stable from ages 8 to 13 years for all 4 groups in crude and adjusted analyses (eTable 3 in Supplement 1). Notably, all groups had similar trajectories of academic progression with increasing age, overall and for each domain. From ages 8 to 13 years, there was a fixed and stepwise reduction in total mean adjusted score differences when full-term controls were compared with very preterm controls (AMD, −25.2; 95% CI, −30.9 to −19.6), children with low-grade IVH (AMD, −33.3; 95% CI, −45.3 to −21.3), and children with high-grade IVH (AMD, −77.0; 95% CI, −107.0 to −47.0) (eTable 3 in Supplement 1). The performance of all 4 groups of children improved with age fairly consistently (full-term children: adjusted β, 31.4 [95% CI, 31.3-31.4]; very preterm children: adjusted β, 32.3 [95% CI, 31.2-33.5]; children with low-grade IVH: adjusted β, 31.5 [95% CI, 29.0-34.0]; high-grade IVH: adjusted β, 30.2 [95% CI, 24.1-36.4]). The difference in trajectories between groups over time, however, remained fixed, with no significant catch-up, plateau, or decline in total or domain-specific performance with increasing age (eTables 4-8 in Supplement 1).

## Discussion

This study indicated that very preterm children with low-grade (grade 1-2) IVH performed similarly to very preterm children without IVH at school. However, children with grade 2 IVH showed poorer performance. Most children with low-grade IVH successfully passed national assessments at ages 8 to 9, 10 to 11, and 12 to 13 years, showing no significant decline or catch-up in performance with increasing age. Conversely, children with high-grade IVH (grade 3-4) consistently scored lower than their very preterm counterparts without IVH throughout childhood, especially in numeracy. Nevertheless, most children with high-grade IVH passed assessments at age 8 to 11 years and showed steady academic growth comparable to other groups.

This study addresses a critical gap in the literature regarding the impact of low-grade IVH on later childhood outcomes, clarifying previously conflicting results.^12,13,14,16,17,20,21^ Our findings suggest that low-grade IVH does not significantly affect school performance; any potential impact appeared to be small and undetectable in this population-based study. However, subgroup analyses revealed that grade 2, but not grade 1, IVH was associated with worse school outcomes at age 8 to 9 years. The small but meaningful effect size for grade 2 IVH highlighted the large sample size needed to detect differences and may explain inconsistencies in prior studies that aggregated IVH grades 1 and 2.^12,13,14,16,17,20,21^ Of note, our findings align with a recent UK study that reported no adverse effects of grade 1 IVH on neurodevelopment at age 2 years but increasing impacts of grades 2 through 4.^9^ Furthermore, there was no association between low-grade IVH and school performance after adjusting for confounders. Previous studies could not adjust for this range of confounders, predisposing the findings to residual confounding and conflicting results.^12,13,14,16,17,20,21^

Existing literature consistently indicates that infants with high-grade IVH face poor outcomes.^18^ Our study contributes to this understanding by demonstrating that even within a contemporary cohort, surviving children with high-grade IVH performed significantly worse academically throughout childhood compared with children born very preterm without IVH. Notably, most very preterm survivors with high-grade IVH successfully passed their school assessments at age 8 to 11 years and continued to develop academically over time, a novel finding.

While it is well established that very preterm children generally exhibit lower cognitive scores and diminished academic performance compared with their term-born peers,^25,26^ the specific impact of IVH has not been studied previously to our knowledge. Our study found that infants with high-grade IVH maintained a fixed academic performance deficit compared with both very preterm and full-term controls throughout childhood, indicating no significant decline or catch-up with age.

Our findings regarding the minimal impact of low-grade IVH, the potential impact of IVH of grade 2, and trajectories following brain injury provide crucial long-term prognostic insights for discussion with families. Previous research in the preterm population highlighted challenges related to attention, executive function, and numeracy skills at school age, often without considering brain injuries.^25,27,28^

Our study found that children with high-grade IVH exhibited poorer performance in numeracy compared with very preterm controls, indicating further susceptibility in this domain that may benefit from targeted support. This is important information for families and educators. Existing initiatives such as preterm-awareness education packages for schools, deferred school entry, and personalized interventions targeting weaknesses in learning domains may help this population.^29,30^

Amid the increasing resuscitation of preterm neonates born at 22 to 23 weeks of gestation, who face the highest risk for IVH, and the stagnation of IVH rates internationally, further large-scale population studies are essential to assess the impact of specific IVH grades and their laterality on childhood outcomes.^5,9,10,31,32^ This would provide more personalized prognostic information for families and enhance our understanding of how these injuries affect children across the spectrum of severity and prematurity. Future research should also investigate the influence of co-occurring morbidities, disabilities, and behavioral issues on school performance.

### Strengths and Limitations

A key strength of this research is its population-based design, enhancing generalizability and reducing selection bias by including hard-to-reach participants. The use of national standardized education data, along with both very preterm and full-term controls, provides a measure of children’s academic performance compared with peers while accounting for environmental factors.

However, this study also has limitations. We could not disaggregate results by IVH laterality or explore coexisting white matter injury or complications like posthemorrhagic hydrocephalus due to unavailable data. IVH was identified via cranial ultrasonography instead of magnetic resonance imaging, and while studies show good interrater reliability for identifying high-grade IVH via ultrasonography, reliability is lower for less-severe grades.^6,33,34^ Given a prespecified primary outcome and that the domain-specific analyses were correlated exploratory secondary outcomes, we did not apply multiple-comparison adjustments.^35^ Very preterm children with chronic health issues may have poorer school attendance, potentially impacting academic performance; we were unable to explore this effect modifier. We also did not have access to data on individualized educational supports, which may influence both academic outcomes and school progression. Additionally, standardized assessments like NAPLAN may not fully capture the progress of children receiving specialized support.

## Conclusions

In this cohort study of school performance following IVH, we found that low-grade IVH was not associated with poorer school performance, while grade 2 IVH was associated with reduced performance. Children with high-grade IVH consistently performed worse than their peers born very preterm throughout childhood. However, all groups—children born at full term and very preterm children without IVH, with low-grade IVH, and with high-grade IVH—showed parallel academic growth trajectories. These findings emphasize the need for ongoing support to help children with IVH achieve their full academic potential.

## Notes

Supplement 1. **eTable 1.** Overview of Included Databases **eFigure 1.** Directed Acyclic Graph **eFigure 2.** Flow Diagram **eTable 2.** Sensitivity Analysis of Attainment Scores at 8-9 Years, Comparing Results With and Without Imputed Missing Values **eTable 3.** Growth Modeling of Mean Total Academic Score Trajectories by Group **eTable 4.** Growth Modeling of Mean Reading Academic Score Trajectories by Group **eTable 5.** Growth Modeling of Mean Writing Academic Score Trajectories by Group **eTable 6.** Growth Modeling of Mean Spelling Academic Score Trajectories by Group **eTable 7.** Growth Modeling of Mean Grammar Academic Score Trajectories by Group **eTable 8.** Growth Modeling of Mean Numeracy Academic Score Trajectories by Group **eTable 9.** Sensitivity Analysis Using Linear and Logistic Regression to Assess NAPLAN Performance at 8 to 9 Years of Age by Individual Grade of IVH **eTable 10.** Logistic Regression of School Performance Above National Minimum Standards at Age 10 to 11 Years and 12 to 13 Years Among Children With High- and Low-Grade IVH Compared With Preterm-Born Controls

Supplement 2.
Data Sharing Statement
