# Supplementary material for: School Performance of Preterm-Born Children After Intraventricular Hemorrhage
Source: JAMA Netw Open. 2025 Dec 11;8(12):e2547584. doi: 10.1001/jamanetworkopen.2025.47584 (PMC12699354; doi:10.1001/jamanetworkopen.2025.47584)
Supplement: Supplement 1. — eTable 1. Overview of Included Databases eFigure 1. Directed Acyclic Graph eFigure 2. Flow Diagram eTable 2. Sensitivity Analysis of Attainment Scores at 8-9 Years, Comparing Results With and Without Imputed Missing Values eTable 3. Growth Modeling of Mean Total Academic Score Trajectories by Group eTable 4. Growth Modeling of Mean Reading Academic Score Trajectories by Group eTable 5. Growth Modeling of Mean Writing Academic Score Trajectories by Group eTable 6. Growth Modeling of Mean Spelling Academic Score Trajectories by Group eTable 7. Growth Modeling of Mean Grammar Academic Score Trajectories by Group eTable 8. Growth Modeling of Mean Numeracy Academic Score Trajectories by Group eTable 9. Sensitivity Analysis Using Linear and Logistic Regression to Assess NAPLAN Performance at 8 to 9 Years of Age by Individual Grade of IVH eTable 10. Logistic Regression of School Performance Above National Minimum Standards at Age 10 to 11 Years and 12 to 13 Years Among Children With High- and Low-Grade IVH Compared With Preterm-Born Controls [file jamanetwopen-e2547584-s001.pdf]

Supplementary Online Content

Rees P, Dronavalli M, Carter B, et al. School performance of preterm-born children after intraventricular hemorrhage. *JAMA Netw Open*. 2025;8(12):e2547584. doi:10.1001/jamanetworkopen.2025.47584

eTable 1. Overview of Included Databases

eFigure 1. Directed Acyclic Graph

eFigure 2. Flow Diagram

eTable 2. Sensitivity Analysis of Attainment Scores at 8-9 Years, Comparing Results With and Without Imputed Missing Values

eTable 3. Growth Modeling of Mean Total Academic Score Trajectories by Group

eTable 4. Growth Modeling of Mean Reading Academic Score Trajectories by Group

eTable 5. Growth Modeling of Mean Writing Academic Score Trajectories by Group

eTable 6. Growth Modeling of Mean Spelling Academic Score Trajectories by Group

eTable 7. Growth Modeling of Mean Grammar Academic Score Trajectories by Group

eTable 8. Growth Modeling of Mean Numeracy Academic Score Trajectories by Group

eTable 9. Sensitivity Analysis Using Linear and Logistic Regression to Assess NAPLAN Performance at 8 to 9 Years of Age by Individual Grade of IVH

eTable 10. Logistic Regression of School Performance Above National Minimum Standards at Age 10 to 11 Years and 12 to 13 Years Among Children With High- and Low-Grade IVH Compared With Preterm-Born Controls

This supplementary material has been provided by the authors to give readers additional information about their work.

© 2025 Rees P et al. *JAMA Network Open*.

eTable 1: Overview of included databases	
**Database**	**Description**
Neonatal Intensive Care Units (NICU) Data Collection	This dataset includes demographic, clinical, and treatment information for infants admitted to neonatal units in New South Wales within the first 28 days of life, specifically for those born before 32 weeks of gestation.
New South Wales Perinatal Data Collection (NSW PDC)	This collection provides maternal and infant birth data for every infant born in New South Wales, helping to identify infants who were born in NSW specifically and a term control cohort.
Admitted Patient Data Collection (APDC)	This database documents all hospital admissions (including discharges, transfers, and mortality) for residents of New South Wales. It was used to identify infants who had died and to gather additional covariate data.
Australian Bureau of Statistics Cause of Death	This dataset outlines the causes of death for residents in New South Wales and was used alongside the APDC to exclude infants who had died.
National Assessment Program Literacy and Numeracy (NAPLAN) Database	This database contains information on child and parental characteristics as well as results from the standardised NAPLAN assessments conducted at grades 3 (ages 8-9), 5 (10-11), 7 (12-13), and 9 (14-15). Introduced in 2008, NAPLAN is compulsory for all Australian schools, assessing skills in reading, writing, spelling, grammar, and numeracy. Each assessment is scored from 0 to 1,000, with scores standardised across grades to ensure consistency. A National Minimum Standard (NMS) is established for each grade; those not participating in NAPLAN are considered to not meet the NMS.

© 2025 Rees P et al. *JAMA Network Open*.

eFigure 1: Directed acyclic graph illustrating the key assumed causal relationships between variables in relation to the exposure (IVH) and the outcome (educational performance). Social factors included rural residence, socioeconomic status, parental education, maternal age and Indigenous status. Confounding was addressed via regression adjustment.

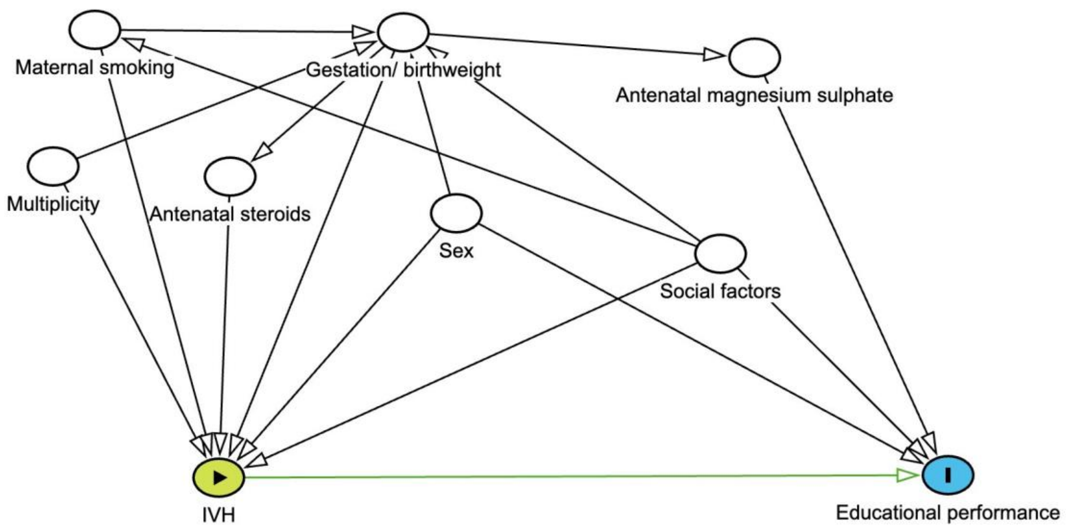

© 2025 Rees P et al. *JAMA Network Open*.

eFigure 2: Flow diagram

Very preterm infants <32 weeks' gestation

- High-grade IVH (n=252)
- Low-grade IVH (n=962)
- No IVH (n=4,267)

Term controls ≥37 weeks' gestation not admitted to a New South Wales neonatal unit

(n=622, 351)

Deaths before NAPLAN grade 3

- High-grade IVH (n=123)
- Low-grade IVH (n=78)
- Preterm controls (n=156)
- Term controls (n=905)

No NAPLAN record (E.g. missing due to 2020 cancellation or born late in the Epoch and not eligible for 2021 test)

- High-grade IVH (n=44)
- Low-grade IVH (n=327)
- Preterm controls (n=1,554)
- Term controls (n=216,456)

NAPLAN data available 2016-2019 and 2021

- High-grade IVH (n=85)
- Low-grade IVH (n=557)
- Preterm controls (n=2,557)
- Term controls (n=404,990)

© 2025 Rees P et al. *JAMA Network Open*.

eTable 2: Sensitivity analysis demonstrating attainment scores at 8-9 years for each group with and without imputing missing values for those who did not meet NMS (adjusted for gestation, birth weight z-score, sex, receipt of antenatal magnesium sulphate, receipt of antenatal steroids, multiplicity, Indigenous status, ARIA score (rurality), socioeconomic status, maternal age, maternal smoking in pregnancy, parental education, test year, age at test)							
Outcome	Preterm controls<br><br>N=2525	Low-grade IVH<br><br>N=552	High-grade IVH<br><br>n=85	MD (95% CI)	aMD (95% CI)	MD (95% CI)	aMD (95% CI)
				Low-grade IVH		High-grade IVH	
Total Z score (missing values imputed)	0.04 (1)	-0.09 (1.1)	-0.52 (1.2)	-0.13 (-0.22, -0.04)	-0.06 (-0.14, 0.03)	-0.56 (-0.77, -0.35)	-0.50 (-0.71, -0.30)
	N=2371	N=501	n=67				
Total Z-score (without imputing missing values)	0.02 (1)	-0.06 (1.0)	-0.25 (1.0)	-0.08 (-0.17, 0.02)	-0.01 (-0.1, 0.1)	-0.26 (-0.5, -0.02)	-0.19 (-0.42, 0.04)

© 2025 Rees P et al. *JAMA Network Open*.

eTable 3: Growth modeling of mean total academic score trajectories by group (adjusted for, sex, multiplicity, Indigenous status, ARIA score (rurality), socioeconomic status, maternal age, maternal smoking in pregnancy, parental education)						
Parameters and growth predictors	Crude coefficient (SE)	95% CI	p-value	Adjusted coefficient (SE)	95% CI	p-value
Fixed difference						
Term controls	- Reference -					
Preterm controls	-37.3 (2.8)	-42.9, -31.7	<0.001	-25.2 (2.9)	-30.9, -19.6	<0.001
Low-grade IVH	-48.5 (6.2)	-60.6, -36.4	<0.001	-33.3 (6.3)	-45.3, -21.3	<0.001
High-grade IVH	-93.7 (15.1)	-123.2, -64.1	<0.001	-77.0 (15.4)	-107.0 -47.0	<0.001
Trajectory (gradient/ slope between grade 3-7)						
Term controls	31.4 (0.04)	31.3, 31.4	<0.001	31.4 (0.04)	31.3, 31.4	<0.001
Preterm controls	32.3 (0.6)	31.1, 33.4	<0.001	32.3 (0.6)	31.2, 33.5	<0.001
Low-grade IVH	31.5 (1.3)	29.0, 34.0	<0.001	31.5 (1.3)	29.0, 34.0	<0.001
High-grade IVH	32.1 (3.0)	26.3, 37.9	<0.001	30.2 (3.1)	24.1, 36.4	<0.001
Grades # IVH (gradient/ slope between grade 3 to 7 relative to controls)						
Term controls	- Reference -					
Preterm controls	0.9 (0.6)	-0.2, 2.0	0.117	1.0 (0.6)	-0.2, 2.1	0.092
Low-grade IVH	0.2 (1.3)	-2.4, 2.7	0.9	0.14 (1.3)	-2.4, 2.7	0.913
High-grade IVH	0.8 (3.0)	-5.1, 6.6	0.26	-1.12 (3.1)	-7.2, 5.0	0.72

© 2025 Rees P et al. *JAMA Network Open*.

eTable 4: Growth modeling of mean reading academic score trajectories by group (adjusted for, sex, multiplicity, Indigenous status, ARIA score (rurality), socioeconomic status, maternal age, maternal smoking in pregnancy, parental education)						
Parameters and growth predictors	Crude coefficient (SE)	95% CI	p-value	Adjusted coefficient (SE)	95% CI	p-value
Fixed difference						
Term controls	- Reference -					
Preterm controls	-31.1 (3.8)	-38.6, -23.6	<0.001	-17.1 (3.9)	-24.7, -9.5	<0.001
Low-grade IVH	-41.4 (8.3)	-57.7, -25.1	<0.001	-24.0 (8.3)	-40.3, -7.7	<0.001
High-grade IVH	-81.0 (20.3)	-120.7, -41.3	<0.001	-69.2 (20.8)	-110.0, -28.4	<0.001
Trajectory (gradient/ slope between 8-13 years; grade 3-7)						
Term controls	31.1 (0.1)	31.0, 31.2	<0.001	31.1 (0.1)	30.9, 31.2	<0.001
Preterm controls	31.6 (0.8)	30.0, 33.2	<0.001	31.4 (0.8)	29.8, 33.1	<0.001
Low-grade IVH	30.4 (1.8)	26.8, 34.0	<0.001	30.3 (1.8)	26.7, 33.9	<0.001
High-grade IVH	28.1 (4.2)	19.8, 36.4	<0.001	27.8 (4.5)	19.0, 36.5	<0.001
Grades # IVH (gradient/ slope between 8-13 years; grade 3-7 relative to controls)						
Term controls	- Reference -					
Preterm controls	0.5 (0.82)	-1.1 2.1	0.573	0.4 (0.8)	-1.3, 2.0	0.649
Low-grade IVH	-0.7(1.8)	-4.3, 2.9	0.703	-0.7 (1.8)	-4.3, 2.9	0.692
High-grade IVH	-3.0 (4.2)	-11.3, 5.3	0.484	-3.3 (4.5)	-12.0, 5.5	0.460

© 2025 Rees P et al. *JAMA Network Open*.

eTable 5: Growth modeling of mean writing academic score trajectories by group (adjusted for, sex, multiplicity, Indigenous status, ARIA score (rurality), socioeconomic status, maternal age, maternal smoking in pregnancy, parental education)						
Parameters and growth predictors	Crude coefficient (SE)	95% CI	p-value	Adjusted coefficient (SE)	95% CI	p-value
Fixed difference						
Term controls	- Reference -					
Preterm controls	-34.5 (3.3)	-40.9, -28.0	<0.001	-26.1 (3.4)	-32.7, -19.5	<0.001
Low-grade IVH	-44.3 (7.2)	-58.4, -30.2	<0.001	-32.6 (7.2)	-46.8, -18.5	<0.001
High-grade IVH	-87.5 (17.4)	-121.6, -53.4	<0.001	-69.0 (17.9)	-104.1, -33.8	<0.001
Trajectory (gradient/ slope between 8-13 years; grade 3-7)						
Term controls	25.8 (0.06)	25.7, 25.9	<0.001	25.9 (0.06)	25.8, 26.0	<0.001
Preterm controls	25.9 (0.8)	24.3, 27.4	<0.001	26.4 (0.8)	24.9. 28.0	<0.001
Low-grade IVH	25.5 (1.7)	22.1, 29.0	<0.001	25.8 (1.7)	22.4, 29.2	<0.001
High-grade IVH	24.9 (4.0)	16.9, 32.8	<0.001	22.5 (4.2)	14.3, 30.8	<0.001
Grades # IVH (gradient/ slope between 8-13 years; grade 3-7 relative to controls)						
Term controls	- Reference -					
Preterm controls	0.1 (0.8)	-1.5, 1.6	0.942	0.5 (0.8)	-1.0, 2.1	0.493
Low-grade IVH	-0.3 (1.7)	-3.7, 3.2	0.885	-0.1 (1.7)	-3.5, 3.3	0.956
High-grade IVH	-0.9 (4.0)	-8.9, 7.0	0.818	-3.4 (4.2)	-11.6, 4.9	0.422

© 2025 Rees P et al. *JAMA Network Open*.

eTable 6: Growth modeling of mean spelling academic score trajectories by group (adjusted for, sex, multiplicity, Indigenous status, ARIA score (rurality), socioeconomic status, maternal age, maternal smoking in pregnancy, parental education)						
Parameters and growth predictors	Crude coefficient (SE)	95% CI	p-value	Adjusted coefficient (SE)	95% CI	p-value
Fixed difference						
Term controls	- Reference -					
Preterm controls	-39.6 (2.7)	-45.0, -34.3	<0.001	-29.2 (2.8)	-34.6, -23.7	<0.001
Low-grade IVH	-46.1 (5.9)	-57.6, -34.5	<0.001	-32.1 (5.9)	-43.7, -20.5	<0.001
High-grade IVH	-90.9 (14.5)	-119.3, -62.5	<0.001	-80.9 (14.8)	-109.9, -51.9	<0.001
Trajectory (gradient/ slope between 8-13 years; grade 3-7)						
Term controls	34.9 (0.04)	34.9, 35.0	<0.001	34.9 (0.04)	34.8, 34.9	<0.001
Preterm controls	38.0 (0.5)	36.0, 39.0	<0.001	38.1 (0.5)	37.0. 39.2	<0.001
Low-grade IVH	36.6 (1.2)	34.3, 39.0	<0.001	36.4 (1.2)	34.0, 38.7	<0.001
High-grade IVH	39.6 (2.8)	34.2, 45.0	<0.001	38.7 (2.9)	33.0, 44.4	<0.001
Grades # IVH (gradient/ slope between 8-13 years; grade 3-7relative to controls)						
Term controls	- Reference -					
Preterm controls	3.0 (0.5)	2.0, 4.1	<0.000	3.2 (0.5)	2.2, 4.3	<0.000
Low-grade IVH	1.7 (1.2)	-0.7, 4.0	0.162	1.5 (1.2)	-0.9, 3.9	0.217
High-grade IVH	4.6 (2.8)	-0.8, 10.0	0.093	3.9 (2.9)	-1.8, 9.6	0.184

© 2025 Rees P et al. *JAMA Network Open*.

eTable 7: Growth modeling of mean grammar academic score trajectories by group (adjusted for, sex, multiplicity, Indigenous status, ARIA score (rurality), socioeconomic status, maternal age, maternal smoking in pregnancy, parental education)						
Parameters and growth predictors	Crude coefficient (SE)	95% CI	p-value	Adjusted coefficient (SE)	95% CI	p-value
Fixed difference						
Term controls	- Reference -					
Preterm controls	-46.4 (4.1)	-54.5, -38.4	<0.001	-31.7 (4.2)	-39.9, -23.5	<0.001
Low-grade IVH	-63.8 (8.9)	-81.3, -46.3	<0.001	-46.1 (9.0)	-63.7, -28.6	<0.001
High-grade IVH	-121.4 (21.8)	-164.2, -78.8	<0.001	-98.3 (22.4)	-142.2, -54.5	<0.001
Trajectory (gradient/ slope between 8-13 years; grade 3-7)						
Term controls	26.2 (0.1)	26.1, 26.3	<0.001	26.3 (0.1)	26.1, 26.4	<0.001
Preterm controls	27.8 (0.9)	26.1, 29.6	<0.001	27.8 (0.9)	26.0, 29.6	<0.001
Low-grade IVH	27.4 (2.0)	23.5, 31.3	<0.001	27.6 (2.0)	23.7, 31.5	<0.001
High-grade IVH	30.0 (4.6)	21.0, 39.0	<0.001	27.7 (4.8)	18.3, 37.2	<0.001
Grades # IVH (gradient/ slope between 8-13 years; grade 3-7 relative to controls)						
Term controls	- Reference -					
Preterm controls	1.6 (0.9)	-0.1, 3.4	0.067	1.6 (0.9)	-0.22, 3.3	0.086
Low-grade IVH	1.2 (2.0)	-2.7, 5.1	0.545	1.3 (2.0)	-2.6, 5.2	0.507
High-grade IVH	3.8 (4.6)	-5.2, 12.8	0.413	1.5 (4.8)	-8.0, 11.0	0.760

© 2025 Rees P et al. *JAMA Network Open*.

eTable 8: Growth modeling of mean numeracy academic score trajectories by group (adjusted for, sex, multiplicity, Indigenous status, ARIA score (rurality), maternal smoking, parental education) – not socioeconomic status or maternal age (which was similar between groups) to improve model fit						
Parameters and growth predictors	Crude coefficient (SE)	95% CI	p-value	Adjusted coefficient (SE)	95% CI	p-value
Fixed difference						
Term controls	- Reference -					
Preterm controls	-36.6 (3.5)	-43.4, -29.7	<0.001	-24.5 (3.6)	-32.0, -17.9	<0.001
Low-grade IVH	-48.8 (7.6)	-63.7, -33.9	<0.001	-36.4 (7.7)	-51.4, -21.3	<0.001
High-grade IVH	-89.6 (18.6)	-126.1, -53.2	<0.001	-72.3 (19.2)	-110.0, -34.6	<0.001
Trajectory (gradient/ slope between 8-13 years; grade 3-7)						
Term controls	38.9 (0.1)	38.8, 39.0	<0.001	38.9 (0.1)	38.8, 39.0	<0.001
Preterm controls	38.8 (0.8)	37.3, 40.3	<0.001	38.8 (0.8)	37.3, 40.3	<0.001
Low-grade IVH	38.3 (1.7)	34.9, 41.6	<0.001	38.2 (1.7)	34.9, 41.6	<0.001
High-grade IVH	38.9 (4.0)	31.2, 46.7	<0.001	35.3 (4.2)	27.2, 43.5	<0.001
Grades # IVH (gradient/ slope between 8-13 years; grade 3-7 relative to controls)						
Term controls	- Reference -					
Preterm controls	-0.1 (0.8)	-1.6, 1.4	0.897	-0.1 (0.8)	-1.6, 1.5	0.949
Low-grade IVH	-0.6 (1.7)	-3.9, 2.7	0.715	-0.6 (1.7)	-4.0, 2.7	0.704
High-grade IVH	0.03 (4.0)	-7.7, 7.8	0.995	-3.6 (4.2)	-11.7, 4.6	0.390

© 2025 Rees P et al. *JAMA Network Open*.

eTable 9: Sensitivity analysis using linear and logistic regression to assess NAPLAN performance at 8 to 9 years of age by individual grade of IVH				
Children[a]	MD (95% CI)	AMD (95% CI)[b]	OR (95% CI)	AOR (95% CI)[b]
IVH grade 1 (n = 407)<br>Very preterm controls (n = 2525)	−0.05 (−0.15 to 0.05)	−0.01 (−0.11 to 0.08)	0.86 (0.68 to 1.10)	0.93 (0.72 to 1.22)
IVH grade 2 (n = 145)<br>Very preterm controls (N = 2525)	−0.35 (−0.52 to −0.19)	−0.20 (−0.36 to −0.04)	0.51 (0.36 to 0.72)	0.66 (0.44 to 0.99)
IVH grade 3 (n = 34)<br>Very preterm controls (n = 2525)	−0.17 (−0.50 to 0.16)	−0.18 (−0.50 to 0.13)	0.77 (0.36 to 1.61)	0.74 (0.32 to 1.67)
IVH grade 4 (n = 51)<br>Very preterm controls (n = 2525)	−0.82 (−1.09 to −0.55)	−0.72 (−0.99 to −0.46)	0.31 (0.18 to 0.54)	0.35 (0.19 to 0.67)

Abbreviations: AMD, adjusted mean difference; AOR, adjusted odds ratio; IVH, intraventricular hemorrhage; MD, mean difference; NAPLAN, National Assessment Program—Literacy and Numeracy; NMS, national minimum standards; OR, odds ratio.

[a]Very preterm indicates gestational age less than 32 weeks.

[b]Adjusted for gestation, birth weight *z* score, sex, receipt of antenatal magnesium sulfate, receipt of antenatal steroids, multiples birth, Indigenous status, Accessibility/Remoteness Index of Australia score (rurality), socioeconomic status, maternal age, maternal smoking in pregnancy, parental educational level, test year, and age at test.

© 2025 Rees P et al. *JAMA Network Open*.

eTable 10: logistic regression of school performance above national minimum standards at age 10 to 11 years and 12 to 13 years among children with high- and low-grade IVH compared with preterm-born controls							
NAPLAN domain	Participants, No./total No. (%)			Low-grade IVH		High-grade IVH	
	Very preterm controls[a]	Low-grade IVH	High-grade IVH	OR (95% CI)	AOR (95% CI)[b]	OR (95% CI)	AOR (95% CI)[b]
10-11 y (grade 5)							
Overall	1123/1538 (73.0)	202/297 (68.0)	27/54 (50.0)	0.79 (0.60-1.03)	0.89 (0.65-1.21)	0.37 (0.21-0.64)	0.32 (0.17-0.60)
Reading	1338/1538 (87.0)	240/297 (80.8)	35/54 (64.8)	0.63 (0.45-0.87)	0.68 (0.47-0.98)	0.28 (0.15-0.49)	0.23 (0.12-0.44)
Writing	1279/1538 (83.2)	240/297 (80.8)	37/54 (68.5)	0.85 (0.62-1.17)	1.03 (0.71-1.48)	0.44 (0.24-0.79)	0.42 (0.21-0.81)
Spelling	1346/1538 (87.5)	243/297 (81.8)	37/54 (68.5)	0.64 (0.46-0.89)	0.7 (0.48-1.02)	0.31 (0.17-0.56)	0.29 (0.15-0.56)
Grammar	1291/1538 (83.9)	231/297 (77.8)	39/54 (72.2)	0.67 (0.49-0.91)	0.71 (0.50-1.00)	0.50 (0.27-0.92)	0.55 (0.27-1.08)
Numeracy	1334/1538 (86.7)	238/297 (80.1)	36/54 (66.7)	0.62 (0.45-0.85)	0.73 (0.51-1.05)	0.31 (0.17-0.55)	0.29 (0.15-0.55)
12-13 y (grade 7)							
Overall	380/545 (69.7)	69/109 (63.3)	7/21 (33.3)	0.75 (0.49-1.15)	1.14 (0.68-1.89)	0.22 (0.09-0.55)	0.16 (0.05-0.49)
Reading	466/545 (85.5)	85/109 (78.0)	14/21 (66.7)	0.60 (0.36-1.00)	0.81 (0.45-1.46)	0.34 (0.13-0.87)	0.30 (0.10-0.86)
Writing	452/545 (82.9)	84/109 (77.1)	13/21 (61.9)	0.69 (0.42-1.14)	0.92 (0.52-1.64)	0.33 (0.13-0.83)	0.34 (0.12-1.03)
Spelling	469/545 (86.1)	86/109 (78.9)	15/21 (71.4)	0.61 (0.36-1.02)	0.83 (0.47-1.48)	0.41 (0.15-1.08)	0.30 (0.10-0.88)
Grammar	436/545 (80.0)	79/109 (72.5)	13/21 (61.9)	0.66 (0.41-1.05)	0.91 (0.52-1.57)	0.41 (0.16-1.00)	0.23 (0.08-0.68)
Numeracy	460/545 (84.4)	89/109 (81.7)	10/21 (47.6)	0.82 (0.48-1.41)	1.33 (0.72-2.48)	0.17 (0.07-0.41)	0.13 (0.05-0.37)

Abbreviations: AOR, adjusted odds ratio; IVH, intraventricular hemorrhage; NAPLAN, National Assessment Program—Literacy and Numeracy; OR, odds ratio.

[a]Very preterm indicates gestational age less than 32 weeks.

[b]Adjusted for gestation, birth weight *z* score, sex, receipt of antenatal magnesium sulfate, receipt of antenatal steroids, multiplicity, Indigenous status, Accessibility/Remoteness Index of Australia score (rurality), socioeconomic status, maternal age, maternal smoking in pregnancy, parental educational level, test year, and age at test.

© 2025 Rees P et al. *JAMA Network Open*.
