# Supplementary material for: School Performance of Preterm-Born Children After Intraventricular Hemorrhage
Source: JAMA Netw Open. 2025 Dec 11;8(12):e2547584. doi: 10.1001/jamanetworkopen.2025.47584 (PMC12699354; doi:10.1001/jamanetworkopen.2025.47584)
Supplement: Supplement 2. — Data Sharing Statement [file jamanetwopen-e2547584-s002.pdf]

Data Sharing Statement

Rees. School Performance of Preterm-Born Children After Intraventricular Hemorrhage. *JAMA Netw Open*. Published December 11, 2025. doi:10.1001/jamanetworkopen.2025.47584

Data
Data available: No
